# Immune checkpoint inhibitors plus capecitabine and oxaliplatin in unresectable or advanced biliary tract cancer patients: A retrospective study

**DOI:** 10.3389/fonc.2022.965711

**Published:** 2022-10-06

**Authors:** Jie Zhao, Yongzhong Guo, Wenzhou Ding, Guoyong Han, Chuanwei Jiang, Chao Yang, Yuanchang Hu, Long Zhang, Chen Wu, Ming Ni, Xiangyi Kong, Tian Huang, Chuanyong Zhang, Yongxiang Xia

**Affiliations:** ^1^ Hepatobiliary/Liver Transplantation Center, The First Affiliated Hospital with Nanjing Medical University, Key Laboratory of Living Donor Transplantation, Chinese Academy of Medical Sciences, Nanjing, China; ^2^ Department of General Surgery, Ili & Jiangsu Joint Institute of Health, Ili, China

**Keywords:** immune checkpoint inhibitors, CAPOX, biliary tract cancer, efficacy, safety, prognostic factors

## Abstract

**Objective:**

Immune checkpoint inhibitors (ICIs) have recently been increasingly used in cancer treatment, whereas their clinical application in biliary tract cancer (BTC) patients is uncommon. This study aimed to evaluate the efficacy and safety of ICIs plus capecitabine and oxaliplatin (CAPOX) in the treatment of BTC patients.

**Methods:**

This retrospective study reviewed 26 unresectable or advanced BTC patients who received ICIs plus CAPOX. The treatment continued until disease progression, uncontrollable adverse event (AE) occurrence, intolerable toxicity occurrence, or voluntary withdrawal.

**Results:**

The median treatment cycles were 5.5 [interquartile range (IQR): 3.8–8.0]. Complete response, partial response, stable disease, and progressive disease rates were 0.0%, 46.2%, 23.1%, and 30.8%, respectively. Objective response rate (ORR) and disease control rate (DCR) were 46.2% and 69.2%, correspondingly. Regarding survival, the median progression-free survival (PFS) and overall survival (OS) were 6.1 (95% CI: 4.4–7.7) months and 16.5 (95% CI: 5.0–28.0) months; moreover, the 1-year PFS and OS rates were 21.5% and 54.3%, respectively. An Eastern Cooperative Oncology Group (ECOG) score of 1–3 (vs. 0) was associated with declined DCR, PFS, and OS (all *p* < 0.050). The most common AEs of ICIs plus CAPOX were thrombocytopenia (61.5%), neutropenia (26.9%), and reactive cutaneous capillary endothelial proliferation (RCCEP) (23.1%). Moreover, 13 (50.0%) patients suffered from grade 3–4 AEs, including thrombocytopenia (50.0%), neutropenia (7.7%), liver dysfunction (7.7%), and RCCEP (3.8%). Notably, the majority of AEs were controllable.

**Conclusion:**

ICIs plus CAPOX chemotherapy exhibit a good efficacy and a manageable safety profile in the treatment of patients with unresectable or advanced BTC.

## Introduction

Biliary tract cancer (BTC), originating from biliary tract epithelial cells, is an aggressive malignancy that consists of cholangiocarcinoma (mainly arising in the intrahepatic, perihilar, or distal bile ducts) and gallbladder carcinoma (GBC) (1–3). Regarding the epidemiology of BTC, its incidence and mortality have grown steadily during the past decade, with nearly 210,000 new cases and 174,000 deaths worldwide in 2017 (4–6). Approximately 60%–70% of BTC patients are diagnosed at an advanced stage, missing the surgery timing (7). Subsequently, for those unresectable or metastatic patients, numerous efforts have been made, including chemotherapy, targeted therapy, and percutaneous radiofrequency ablation (8–13). However, only a minority of patients could benefit from the above treatment, and the prognosis of advanced BTC patients is far from satisfactory, with a median overall survival (OS) of 15 months or less (14, 15). Therefore, in order to improve the prognosis, immunotherapy has been applied in BTC treatment in recent years (16, 17).

Immune checkpoint inhibitors (ICIs), a common immunotherapy that prevents immune escape through binding to programmed cell death protein-1 (PD-1) or its ligands (PD-L1/2), are recognized as a crucial milestone owing to their promising efficacy and safety profile in the treatment of solid cancer (18, 19). In order to provide novel treatment options and improve the prognosis, some studies have sought appropriate ICIs to treat BTC patients (20–23). For instance, a previous study showed the modest efficacy of nivolumab in treating refractory BTC patients with an objective response rate (ORR) of 11% and a disease control rate (DCR) of 50%; subsequently, nivolumab was included in National Comprehensive Cancer Network (NCCN) guidelines as a subsequent-line treatment option for disease-progression BTC patients (category 2B) (20). Also, in the KEYNOTE-158 study, advanced BTC patients treated with pembrolizumab treatment had an ORR of 5.8%; meanwhile, the median PFS and OS reached 2.0 months and 7.4 months, respectively (24). Furthermore, another study observed that the median progression-free survival (PFS) and OS were correspondingly 2.9 months and 5.7 months in advanced BTC patients who received nivolumab plus ipilimumab combination treatment (25). The above studies reflect that ICIs provide a particular prospect in treating BTC patients, while the efficacy of ICI alone is not ideal. Of note, the TOPAZ-1 trial utilizes durvalumab (vs. placebo) in combination with gemcitabine plus cisplatin to treat advanced BTC patients, whose ORR reaches 26.7% (vs. 18.7%) and the estimated 2-year OS rate is 24.9% (vs. 10.4%), representing a historical step forward in BTC management (26). Consequently, the combination of ICIs with chemotherapy is considered as a novel idea for advanced BTC treatment, with the potency to change the first-line treatment standard.

Capecitabine plus oxaliplatin (CAPOX) chemotherapy has displayed a non-inferior performance (compared to gemcitabine plus oxaliplatin) in treating BTC patients, with a particular efficacy and manageable safety; meanwhile, the convenient dosing regimen makes it more suitable for the Asian population (27, 28). Therefore, it has been speculated that ICIs combined with CAPOX might be an appropriate therapy for advanced BTC patients. Only one previous study with a relatively small sample size (*N* = 11) explored the efficacy of pembrolizumab plus CAPOX in advanced BTC patients in the United States, whose ORR reached 27.3%; moreover, the median PFS and OS were 4.1 months and 9.9 months, respectively (29). However, the relevant application of ICIs plus CAPOX in treating Chinese BTC patients is still rare, and treatment outcomes need to be further validated in real clinical settings.

Hence, this retrospective study aimed to evaluate the efficacy and safety of ICIs plus CAPOX chemotherapy in treating unresectable or advanced BTC patients.

## Methods

### Patients

This retrospective study reviewed 26 unresectable or advanced BTC patients treated with ICIs plus CAPOX between 24 December 2019, and 6 August 2021. The screening criteria were as follows: (a) diagnosed with unresectable or advanced BTC; (b) aged ≥18 years; (c) received ICIs in combination with CAPOX treatment; and (d) with at least one measurable lesion in line with Response Evaluation Criteria in Solid Tumors (RECIST, version 1.1) (30). Patients who had the following conditions were ineligible for enrollment: (a) had no available data for study analysis; (b) had other primary solid tumors or hematologic malignancies; and (c) were pregnant or breastfeeding. The study was permitted by the Ethics Committee. Additionally, as this was a retrospective study, the Ethics Committee approved waiving the informed consent.

### Data collection

Demographics, disease characteristics, laboratory information, treatment history information, current treatment, treatment response, and adverse events (AEs) of patients were obtained. Treatment response was assessed by imagological examination every 2–3 months per RECIST (version 1.1) (30). Furthermore, follow-up data were also abstracted, with a final follow-up date of 23 January 2022. The median follow-up period was 9.0 months, with a range of 2.1 to 23.4 months.

### Treatment

Patients received ICIs plus CAPOX treatment in a 3-week cycle. ICIs (including camrelizumab, durvalumab, nivolumab, tislelizumab, or sintilimab) were administrated on day 1. The dosage and mode of ICIs were in accordance with the medicine package inserts and the actual patient’s status: camrelizumab (intravenous injection, 200 mg), durvalumab (intravenous injection, 1500 mg), nivolumab (intravenous injection, 3 mg/kg), tislelizumab (intravenous injection, 200 mg), and sintilimab (intravenous injection, 200 mg). Oxaliplatin was administrated by intravenous injection at the dose of 130 mg/m^2^ on day 1, and capecitabine was administrated orally at the dose of 1,000 mg/m^2^ twice a day for 14 days (days 1 to 14). The administration of ICIs plus CAPOX treatment was continued until disease progression, occurrence of uncontrollable AEs, the occurrence of intolerable toxicity, or voluntary discontinuation.

### Assessment

The best response of patients was recorded in the study. ORR and DCR were also calculated. Moreover, time to response (TTR), duration of response (DOR), PFS, and OS were imputed. Additionally, AEs were collected and graded according to Common Terminology Criteria for Adverse Events (CTCAE, version 5.0).

### Statistics

Statistics were performed by SPSS V.22.0 (IBM Corp., USA), and figures were fulfilled by GraphPad Prism V.6.1 (GraphPad Software Inc., USA). Comparison analysis of ORR or DCR was performed using the *χ* ^2^ test or Fisher’s exact test; comparison analysis of TTR or DOR was performed using the Wilcoxon rank sum test or Kruskal–Wallis H rank sum test. The correlations of clinical characteristics with PFS and OS rates were displayed using Kaplan–Meier curves and analyzed by log-rank test. Differences in carbohydrate antigen 199 (CA199) level before and after ICIs plus CAPOX were determined using the Wilcoxon signed-rank test. The correlations of clinical characteristics or the decrease in CA199 with PFS and OS rates was analyzed by log-rank test or Tarone-Ware test, respectively. *p* < 0.05 was considered as significant.

## Results

### Clinical characteristics

The mean age of 26 analyzed BTC patients was 60.3 ± 8.5 years and included 11 (42.3%) women and 15 (57.7%) men (**Table 1**). Among all patients, 7 (26.9%) patients were diagnosed with GBC, while the other 19 (73.1%) patients were identified with intrahepatic cholangiocarcinoma (ICC). Furthermore, 1 (3.8%), 4 (15.4%), 8 (30.8%), 1 (3.8%), and 12 (46.2%) patients were correspondingly assessed for tumor-node-metastasis (TNM) stage IIa, IIIa, IIIb, IIIc, and IV. A total of 19 (73.1%) patients had previous treatment. As to the ICI types, 15 (57.7%), 4 (15.4%), 4 (15.4%), 2 (7.7%), and 1 (3.8%) patient received camrelizumab, durvalumab, nivolumab, tislelizumab, and sintilimab, correspondingly. Additionally, the median treatment period of ICIs plus CAPOX was 5.5 [interquartile range (IQR): 3.8–8.0]. The specific information about the patients’ clinical characteristics is displayed in **Table 1**.

**Table 1:** Clinical characteristics.

Items	BTC patients (*N* = 26)
**Demographics**	
Age (years), mean ± SD	60.3 ± 8.5
Gender, No. (%)	
Female	11 (42.3)
Male	15 (57.7)
**Disease characteristics**	
ECOG score, No. (%)	
0	10 (38.5)
1	13 (50.0)
2	2 (7.7)
3	1 (3.8)
Histological type, No. (%)	
GBC	7 (26.9)
ICC	19 (73.1)
TNM stage, No. (%)	
IIa	1 (3.8)
IIIa	4 (15.4)
IIIb	8 (30.8)
IIIc	1 (3.8)
IV	12 (46.2)
Distant metastasis, No. (%)	24 (92.3)
Lymph node	7 (26.9)
Omentum	5 (19.2)
Liver	4 (15.4)
Lung	4 (15.4)
Bone	2 (7.7)
Enterocoelia	2 (7.7)
Stomach	1 (3.8)
Others	3 (11.5)
**Treatment history**	
Previous treatment, No. (%)	19 (73.1)
Surgery, No. (%)	14 (53.8)
Radical	5 (19.2)
Palliative	7 (26.9)
Biopsy	2 (7.7)
Chemotherapy, No. (%)	10 (38.5)
Immunotherapy^*^, No. (%)	8 (30.8)
Radiotherapy, No. (%)	3 (11.5)
Targeted therapy, No. (%)	2 (7.7)
TACE, No. (%)	1 (3.8)
**Current treatment**	
ICIs, No. (%)	26 (100.0)
Camrelizumab	15 (57.7)
Durvalumab	4 (15.4)
Nivolumab	4 (15.4)
Tislelizumab	2 (7.7)
Sintilimab	1 (3.8)
Chemotherapy of CAPOX, No. (%)	26 (100.0)
Treatment cycles of ICIs plus CAPOX, median (IQR)	5.5 (3.8–8.0)

*Six (23.1%) patients previously received camrelizumab; 1 (3.8%) patient previously received sintilimab; 1 (3.8%) patient previously received tislelizumab. BTC, biliary tract cancer; SD, standard deviation; ECOG, Eastern Cooperative Oncology Group; GBC, gallbladder carcinoma; ICC, intrahepatic cholangiocarcinoma; TNM, tumor–node–metastasis; TACE, transarterial chemoembolization; ICIs, immune checkpoint inhibitors; CAPOX, capecitabine plus oxaliplatin; IQR, interquartile range.

### Best treatment response

**Figure 1:**
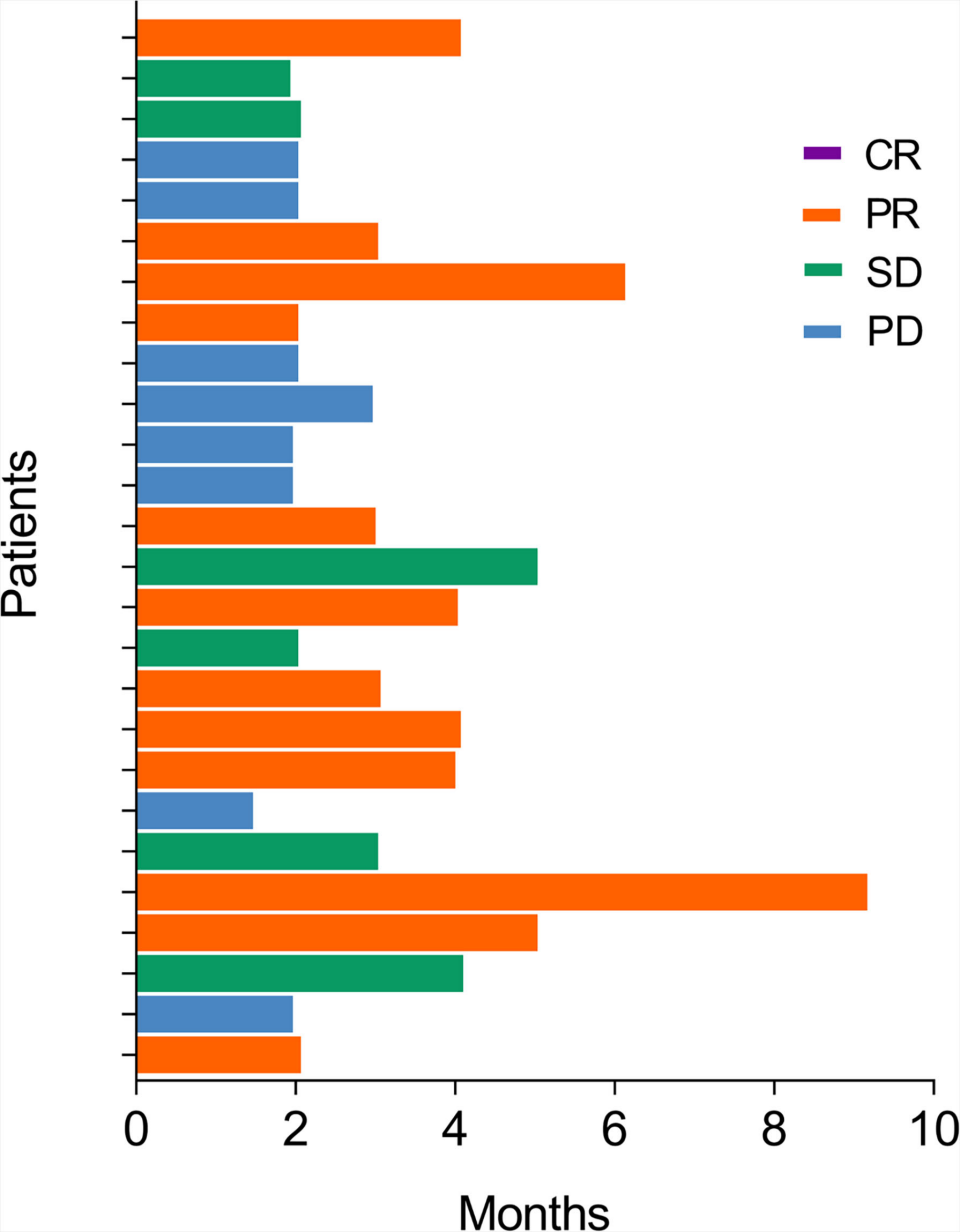
Swimmer plot showing the response status of each BTC patient.

The numbers of cases with complete response (CR), partial response (PR), stable disease (SD), and progressive disease (PD) were 0 (0.0%), 12 (46.2%), 6 (23.1%), and 8 (30.8%), respectively (**Table 2**). Furthermore, ORR and DCR were 46.2% and 69.2%, correspondingly. In addition, the median TTR and DOR were 3.0 (IQR: 2.0–4.1) months and 3.0 (IQR: 2.0–6.8) months. The response status of each patient is displayed in the swimmer plot (**Figure 1**).

**Table 2:** Best treatment response.

Items	BTC patients (*N* = 26)
General response, No. (%)	
CR	0 (0.0)
PR	12 (46.2)
SD	6 (23.1)
PD	8 (30.8)
ORR (CR+PR), No. (%)	12 (46.2)
DCR (CR+PR+SD), No. (%)	18 (69.2)
TTR (months), median (IQR)	3.0 (2.0–4.1)
DOR (months), median (IQR)	3.0 (2.0–6.8)

BTC, biliary tract cancer; CR, complete response, PR, partial response; SD, stable disease; PD, progressive disease; ORR, objective response rate; DCR, disease-control rate; TTR, time to response; IQR, interquartile range; DOR, duration of response.

In terms of the correlation between different patients’ characteristics and optimal treatment response, an Eastern Cooperative Oncology Group (ECOG) score of 1–3 (vs. 0) was associated with declined DCR (50.0% vs. 100.0%, *p* = 0.009); previous chemotherapy yes (vs. no) was associated with prolonged DOR [7.6 (5.5–9.7) months vs. 2.5 (1.2–3.1) months, *p* = 0.011] (**Table 3**). Additionally, different ICI types resulted in different DCR (*p* = 0.040) and TTR (*p* = 0.038). In detail, nivolumab achieved the highest DCR (100.0%), followed by camrelizumab (80.0%) and durvalumab (50.0%). This finding might have been influenced by the relatively small sample size; in detail, only two patients received tislelizumab, and one patient received sintilimab. Meanwhile, nivolumab realized the longest TTR [4.0 (IQR: 3.3–4.1) months], followed by camrelizumab [3.0 (IQR: 2.0–5.0) months] and durvalumab [2.0 (IQR: 2.0–2.0) months]. Furthermore, treatment cycles of ICIs plus CAPOX ≥6 (vs. <6) were associated with an increase in DCR (100% vs. 38.5%, *p* = 0.002) and TTR [4.0 (IQR: 2.1–4.6) months vs. 2.0 (2.0–3.1) months, *p* = 0.019]. Regarding the previous immunotherapy, ORR (55.6% vs. 25.0%, *p* = 0.216) and DCR (77.8% vs. 50.0%, *p* = 0.197) disclosed an increasing trend (lacked statistical significance) in patients without previous immunotherapy compared to patients with previous immunotherapy; TTR [median (IQR): 3.0 (2.0–4.1) months vs. 2.1 (2.0–NA), *p* = 0.823] and DOR [median (IQR): 3.0 (1.8–6.6) months vs. 6.0 (5.0–NA), *p* = 0.281] were not different between patients with and without previous immunotherapy.

**Table 3:** Best treatment response for patients with different characteristics.

Items	ORR No. (%)	DCR No. (%)	TTR (months) median (IQR)	DOR (months) ^*^median (IQR)
ECOG score				
0	5 (50.0)	10 (100.0)	3.5 (2.1–4.3)	5.0 (1.5–9.2)
1–3	7 (43.8)	8 (50.0)	2.1 (2.0–3.8)	3.0 (2.0–6.1)
*p*-value	1.000	**0.009**	0.186	0.289
Histological type				
GBC	5 (71.4)	6 (85.7)	3.0 (2.0–4.0)	3.1 (2.5–7.6)
ICC	7 (36.8)	12 (63.2)	3.0 (2.0–4.1)	3.0 (1.0–7.1)
*p*-value	0.190	0.375	0.862	0.568
TNM stage				
II–III	8 (57.1)	11 (78.6)	3.5 (2.0–4.3)	3.0 (1.2–7.3)
IV	4 (33.3)	7 (58.3)	2.1 (2.0–3.0)	4.6 (2.3–6.8)
*p*-value	0.225	0.401	0.155	0.733
Previous treatment				
No	5 (71.4)	6 (85.7)	3.0 (1.9–4.1)	3.0 (1.0–4.6)
Yes	7 (36.8)	12 (63.2)	2.1 (2.0–4.0)	5.0 (2.0–8.1)
*p*-value	0.190	0.375	0.816	0.289
Previous surgery				
No	5 (41.7)	8 (66.7)	2.5 (2.0–3.8)	3.0 (1.0–4.6)
Yes	7 (50.0)	10 (71.4)	3.1 (2.0–4.3)	5.0 (2.0–8.1)
*p*-value	0.671	1.000	0.326	0.289
Previous chemotherapy				
No	8 (50.0)	12 (75.0)	2.5 (2.0–4.1)	2.5 (1.2–3.1)
Yes	4 (40.0)	6 (60.0)	3.0 (2.0–4.3)	7.6 (5.5–9.7)
*p*-value	0.701	0.664	0.578	**0.011**
Previous immunotherapy				
No	10 (55.6)	14 (77.8)	3.0 (2.0–4.1)	3.0 (1.8–6.6)
Yes	2 (25.0)	4 (50.0)	2.5 (2.0–3.8)	6.0 (5.0– NA)
*p*-value	0.216	0.197	0.823	0.281
Previous radiotherapy				
No	11 (47.8)	15 (65.2)	3.0 (2.0–4.1)	3.0 (2.0–6.1)
Yes	1 (33.3)	3 (100.0)	2.1 (2.0–NA)	NA
*p*-value	1.000	0.529	0.809	0.110
Previous targeted therapy				
No	10 (41.7)	16 (66.7)	3.0 (2.0–4.1)	3.0 (1.8–6.6)
Yes	2 (100.0)	2 (100.0)	5.6 (2.1–NA)	6.0 (5.0–NA)
*p*-value	0.203	1.000	0.310	0.281
Previous TACE				
No	11 (44.0)	17 (68.0)	3.0 (2.0–4.1)	3.1 (2.0–7.1)
Yes	1 (100.0)	1 (100.0)	NA	NA
*p*-value	0.462	1.000	0.461	0.383
ICIs				
Camrelizumab	7 (46.7)	12 (80.0)	3.0 (2.0–5.0)	3.1 (2.0–7.1)
Durvalumab	1 (25.0)	2 (50.0)	2.0 (2.0–2.0)	NA
Nivolumab	4 (100.0)	4 (100.0)	4.0 (3.3–4.1)	4.6 (1.5–9.2)
Tislelizumab	0 (0.0)	0 (0.0)	1.7 (1.5–NA)	NA
Sintilimab	0 (0.0)	0 (0.0)	NA	NA
*p*-value	0.093	**0.040**	**0.038**	0.663
Treatment cycles of ICIs plus CAPOX				
<6	4 (30.8)	5 (38.5)	2.0 (2.0–3.1)	2.5 (0.5–3.1)
≥6	8 (61.5)	13 (100.0)	4.0 (2.1–4.6)	5.6 (2.3–7.8)
*p*-value	0.116	**0.002**	**0.019**	0.125

*Data of 12 patients were available for DOR assessment. ORR, objective response rate; DCR, disease-control rate; TTR, time to response IQR, interquartile range; DOR, duration of response; ECOG, Eastern Cooperative Oncology Group; GBC, gallbladder carcinoma; ICC, intrahepatic cholangiocarcinoma; TNM, tumor–node–metastasis; ICIs, immune checkpoint inhibitors; CAPOX, capecitabine plus oxaliplatin; NA, not available.

The bold values represent the results with statistical significance (P<0.050).

### Survival

**Figure 2:**
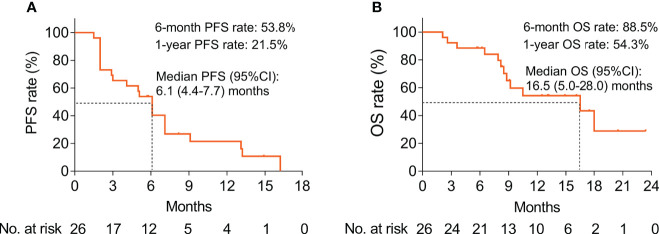
Survival profile of BTC patients treated with ICIs plus CAPOX. Presentation of PFS **(A)** and OS **(B)** by Kaplan–Meier curves in BTC patients.

The median PFS was 6.1 [95% confidence interval (CI): 4.4–7.7] months; the 6-month and 1-year PFS rates were 53.8% and 21.5%, correspondingly (**Figure 2A**). The median OS was 16.5 (95% CI: 5.0–28.0) months, with the respective 6-month and 1-year OS rates of 88.5% and 54.3% (**Figure 2B**).

**Figure 3:**
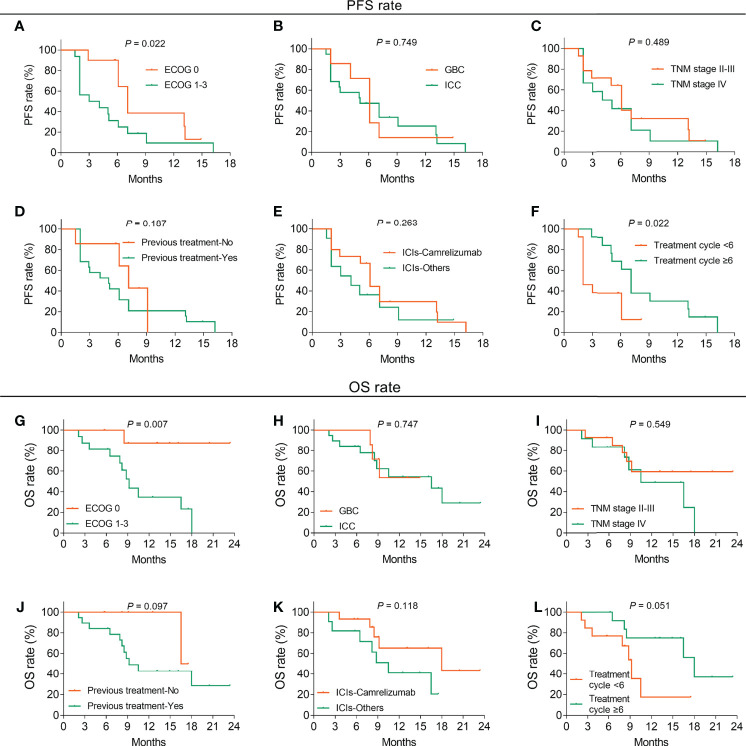
Factors related to PFS and OS in BTC patients. Correlation of ECOG score **(A)**, histological type **(B)**, TNM stage **(C)**, previous treatment **(D)**, ICI types **(E)**, and treatment cycles **(F)** with PFS in BTC patients. Association of ECOG score **(G)**, histological type **(H)**, TNM stage **(I)**, previous treatment **(J)**, ICI types **(K)**, and treatment cycles **(L)** with PFS in BTC patients.

In terms of the relationship between patients’ different characteristics with survival, ECOG 1–3 (vs. 0) (*p* = 0.022) and treatment cycles <6 (vs. ≥6) (*p* = 0.022) were linked to decreased PFS, whereas histological type (*p* = 0.749), TNM stage (*p* = 0.489), previous treatment (*p* = 0.187), and ICIs (*p* = 0.263) were not related to PFS (**Figures 3A–F**). Additionally, ECOG 1–3 (vs. 0) was correlated with shortened OS (*p* = 0.007), while histological type (*p* = 0.747), TNM stage (*p* = 0.549), previous treatment (*p* = 0.097), ICIs (*p* = 0.118), and treatment cycles (*p* = 0.051) were not associated with OS (**Figures 3G–L**).

### AEs

A total of 22 (84.6%) BTC patients experienced AEs, and 13 (50.0%) of these patients suffered from grade 3–4 AEs (**Table 4**). The thrombocytopenia (61.5%), neutropenia (26.9%), reactive cutaneous capillary endothelial proliferation (RCCEP) (23.1%), liver dysfunction (23.1%), hand–foot syndrome (19.2%), and nausea (15.4%) were the most common AEs of ICIs plus CAPOX. Moreover, grade 3–4 AEs included thrombocytopenia (50.0%), neutropenia (7.7%), RCCEP (3.8%), and liver dysfunction (7.7%). Notably, most AEs were controllable. No patients died as a result of AEs.

**Table 4:** AEs of BTC patients.

Items	Total	Grade 1–2	Grade 3–4
Total AEs, No. (%)	22 (84.6)	9 (34.6)	13 (50.0)
Thrombocytopenia, No. (%)	16 (61.5)	3 (11.5)	13 (50.0)
Neutropenia, No. (%)	7 (26.9)	5 (19.2)	2 (7.7)
RCCEP, No. (%)	6 (23.1)	5 (19.2)	1 (3.8)
Liver dysfunction, No. (%)	6 (23.1)	4 (15.4)	2 (7.7)
Hand–foot syndrome, No. (%)	5 (19.2)	5 (19.2)	0 (0.0)
Nausea, No. (%)	4 (15.4)	4 (15.4)	0 (0.0)
Peripheral neuropathy, No. (%)	2 (7.7)	2 (7.7)	0 (0.0)
Vomiting, No. (%)	1 (3.8)	1 (3.8)	0 (0.0)

AEs, adverse events; BTC, biliary tract cancer; RCCEP, reactive cutaneous capillary endothelial proliferation.

### CA199

**Figure 4:**
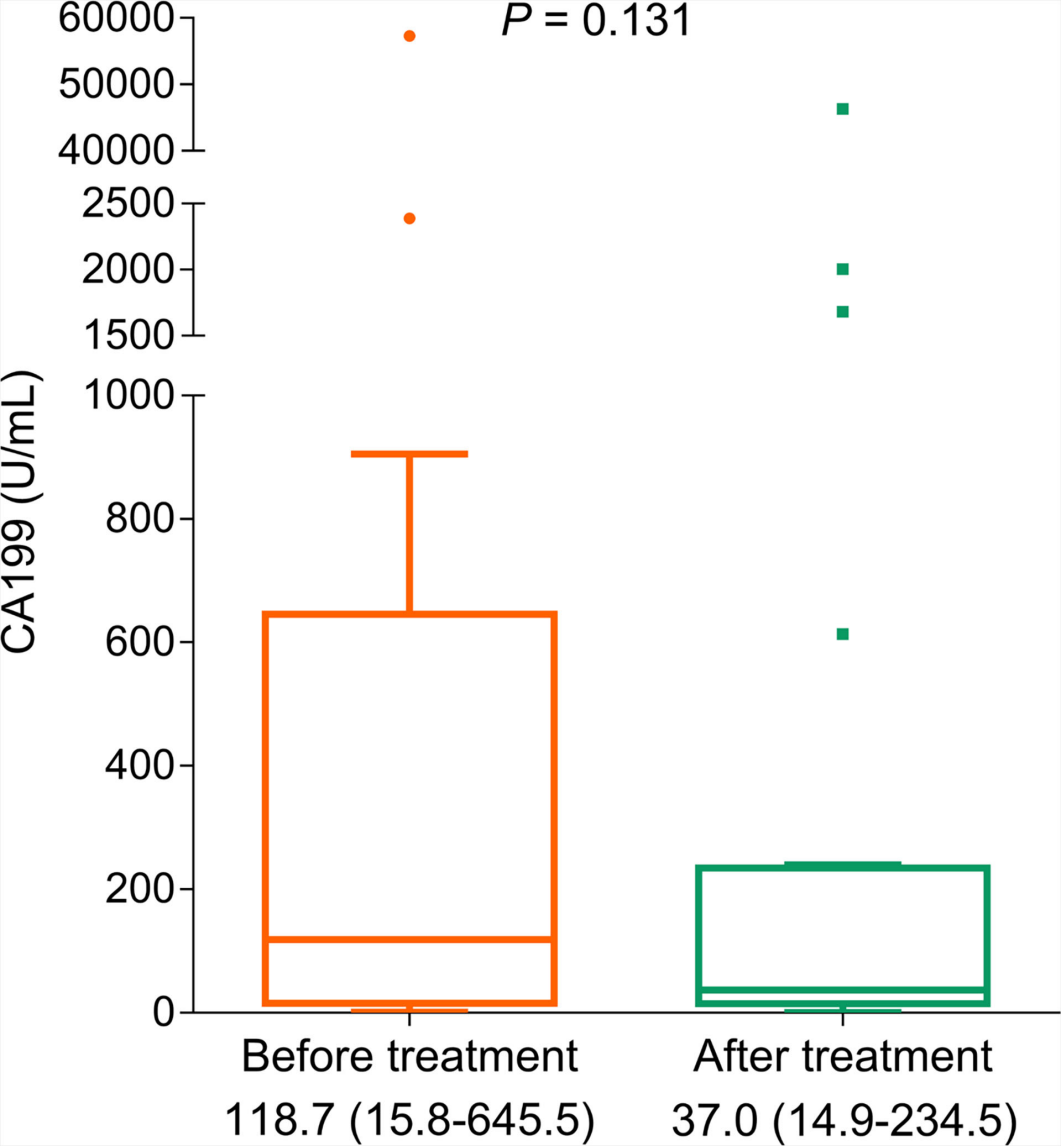
Variation of CA199 before and after treatment in BTC patients.

**Figure 5:**
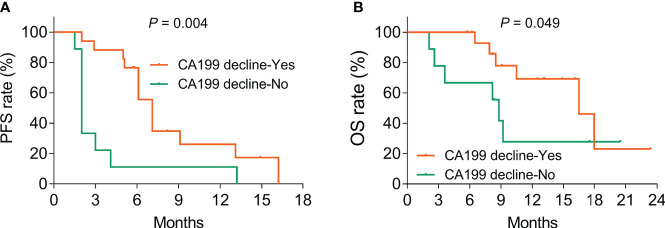
CA199 decline was linked with increased PFS and OS rates in BTC patients. Correlation of CA199 decline with PFS **(A)** and OS **(B)** rates in BTC patients.

The tumor marker CA199 showed a declining trend (without statistical significance) after treatment (*p* = 0.131, **Figure 4**). Furthermore, CA199 decline (vs. no) exhibited an increased PFS rate (*p* = 0.004, **Figure 5A**) and OS rate (*p* = 0.049, **Figure 5B**), suggesting that it could be used as a prognostic biomarker in BTC patients, but this issue needed further exploration.

## Discussion

Some clinical trials have been conducted recently to investigate the efficacy of ICIs plus chemotherapy in advanced BTC patients (21, 31, 32). For instance, the TOPAZ-1 trial finds that durvalumab in combination with gemcitabine plus cisplatin realizes a more delightful treatment response (ORR: 26.7% vs. 18.7%; DCR: 85.3% vs. 82.6%) and survival profile (median PFS: 7.2 months vs. 5.7 months; median OS: 12.8 months vs. 11.5 months) compared to placebo in combination with gemcitabine plus cisplatin in unresectable or metastatic BTC patients (26). Furthermore, a previous study found that the ORR in unresectable or recurrent BTC patients treated with nivolumab plus cisplatin and gemcitabine was 36.7% (31). Another study disclosed that in advanced BTC patients treated with camrelizumab plus gemcitabine and oxaliplatin, the median PFS and OS were 6.1 months and 11.8 months (21). However, the evidence of ICIs’ application combined with CAPOX regimen in BTC patients is still insufficient. The current study disclosed that CR, PR, SD, and PD rates were 0.0%, 46.2%, 23.1%, and 30.8% in BTC patients treated with ICIs plus CAPOX, correspondingly. Furthermore, ORR and DCR were 46.2% and 69.2%, correspondingly. In addition, the median TTR and DOR were 3.0 (IQR: 2.0–4.1) months and 3.0 (IQR: 2.0–6.8) months. The findings reflected a certain efficacy of ICIs plus CAPOX chemotherapy in treating unresectable or advanced BTC patients. Additionally, the different clinical outcomes between the current study and TOPAZ-1 trial might be as follows: The ICIs utilized in this study contained PD-1 inhibitor (including nivolumab, pembrolizumab, camrelizumab, etc.) and programmed cell death ligand-1 (PD-L1) (durvalumab) as well, while the TOPAZ-1 trial only used durvalumab (vs. placebo). The different molecular structures and targets between PD-1 and PD-L1 inhibitors might lead to the different clinical outcomes (33). However, the detailed comparison of efficacy and safety between PD-1 and PD-L1 inhibitors in treating BTC patients needed further validation.

Regarding the efficacy of chemotherapy alone in treating BTC patients, the ABC-02 study demonstrates that median PFS and OS reach 8.0 months and 11.7 months in advanced BTC patients treated with cisplatin plus gemcitabine, respectively (34). Another study finds that advanced BTC patients who received CAPOX treatment had a median PFS and OS of 15.4 weeks and 32.7 weeks, respectively (27), whereas in the current study, the median PFS and OS of BTC patients who received ICIs plus CAPOX were 6.1 (95% CI: 4.4–7.7) months and 16.5 (95% CI: 5.0–28.0) months, respectively; meanwhile, their 1-year PFS and OS rates were correspondingly 21.5% and 54.3%. Clinically speaking, the outcomes of ICIs plus chemotherapy were more delightful than those in the chemotherapy alone (27, 34). The potential causes might be as follows: (1) ICIs plus CAPOX chemotherapy might lead to increased immune recognition and modified immunosuppressive tumor microenvironment (TME) (35). (2) A potential synergy was observed between ICIs and CAPOX, which enhanced the capability of the immune system to recognize and eradicate the tumor cells (36). As a result, the anti-tumor immune response was established, and patients who received ICIs plus CAPOX chemotherapy had longer long-term survival than those who received chemotherapy alone.

Aside from using ICIs plus chemotherapy, some studies attempt to apply ICIs plus targeted agents or combination immunotherapy in the treatment of advanced BTC patients (25, 37). For instance, a previous study used pembrolizumab plus lenvatinib to treat refractory BTC patients, and the median PFS and OS were 4.9 months and 11.0 months, respectively (37). Another study found that in advanced BTC patients treated with the combination of nivolumab and ipilimumab immunotherapy, the corresponding ORR and DCR were 23% and 44%; meanwhile, the median PFS and OS were 2.9 months and 5.7 months, respectively (25). In contrast, the outcomes of the above studies are inferior compared to the current study, which implies that chemotherapy is essential in BTC treatment.

Additionally, this study also revealed that ECOG score 1–3 (vs. 0) was associated with shorter PFS and OS, while treatment cycles ≥6 (vs. <6) were associated with longer PFS in BTC patients. Possible explanations might be as follows: (1) Elevated ECOG score represented inferior physical status; subsequently, the survival profile of high-ECOG score BTC patients treated with ICIs plus CAPOX would be weakened (38). (2) Patients would benefit more from a sustained treatment regimen; therefore, treatment cycles ≥6 (vs. <6) were associated with prolonged PFS in BTC patients. Also, the present study found that ORR and DCR disclosed an increasing trend (but lacked statistical significance) in patients without previous immunotherapy compared to patients with previous immunotherapy, which might be interfered by the small sample size. Consequently, the correlation of previous immunotherapy with treatment response in BTC patients needed further exploration.

According to the previous studies, the incidence of grade 3–4 AEs in advanced BTC patients treated with ICIs plus chemotherapy is nearly 53%–90%; additionally, the most common grade 3–4 AEs include white blood cell decline (17.4%–43.0%), platelet count decline (12.0%–56.2%), and neutrophil count decline (25.0%–70.0%) (31, 32, 39). In this study, a total of 22 (84.6%) BTC patients experienced AEs, with 13 (50.0%) patients suffering from grade 3–4 AEs, with the most common grade 3–4 AEs of thrombocytopenia (50.0%) and neutropenia (7.7%). The AEs were manageable and resolved with appropriate treatment, and no patients died as a result of AEs. These findings suggested that ICIs plus CAPOX was a well-tolerant treatment choice for patients with unresectable or advanced BTC. Moreover, concerning the safety of different chemotherapy regimens, it is reported that the incidence of grade 3–4 AEs of CAPOX is relatively lower compared to gemcitabine plus oxaliplatin/cisplatin in treating advanced BTC patients, implying a relatively better safety profile of CAPOX compared to gemcitabine-based chemotherapy (27, 28).

The current study reflected the efficacy and safety of ICIs plus CAPOX applied to unresectable or advanced BTC patients, whereas some limitations existed in this study. Firstly, the sample size of this study was relatively small, which might weaken the statistical power. Therefore, studies with a large sample size were needed to further validate the findings. Secondly, this was a retrospective study, subsequently, the selection bias was difficult to avoid. Thirdly, the current study did not recruit a control cohort, which was required for further study. Fourthly, this was a single-center study conducted in China; consequently, further multi-center studies should be carried out to lessen the selective bias.

In conclusion, ICIs plus CAPOX chemotherapy exhibit a good efficacy and a manageable safety profile in treating unresectable or advanced BTC patients, but large-scale studies are needed to validate it.

## Data availability statement

The original contributions presented in the study are included in the article/Supplementary Material. Further inquiries can be directed to the corresponding authors.

## Ethics statement

The studies involving human participants were reviewed and approved by Ethics Committee of The First Affiliated Hospital with Nanjing Medical University. Written informed consent for participation was not required for this study in accordance with the national legislation and the institutional requirements.

## Author contributions

YX and CZ substantially contributed to the conception and the design of the study. JZ, YG, and WD were responsible for the acquisition of the data. GH, CJ, and CY were responsible for the analysis of the data. YH, LZ, and CW contributed to the interpretation of the data. MN, XK, and TH contributed to manuscript drafting and edited English language. All authors have read and approved the final manuscript.

## Funding

This study was supported by the National Natural Science Foundation of China (No. 82070676).

## Conflict of interest

The authors declare that the research was conducted in the absence of any commercial or financial relationships that could be construed as a potential conflict of interest.

## Publisher’s note

All claims expressed in this article are solely those of the authors and do not necessarily represent those of their affiliated organizations, or those of the publisher, the editors and the reviewers. Any product that may be evaluated in this article, or claim that may be made by its manufacturer, is not guaranteed or endorsed by the publisher.
